# An Atypical Presentation of Thyroid Eye Disease in a Patient With a Remote History of Graves’ Disease

**DOI:** 10.7759/cureus.86621

**Published:** 2025-06-23

**Authors:** Nancy L Van Buren, Caroline Y Yu, Elizabeth A Bradley

**Affiliations:** 1 Medical Affairs, Innovative Blood Resources, New York Blood Center Enterprises, St. Paul, USA; 2 Ophthalmology, Mayo Clinic, Rochester, USA

**Keywords:** atypical presentation, delayed onset ted, graves’ disease, reactivation, thyroid eye disease, thyrotropin receptor antibodies (trab)

## Abstract

Thyroid eye disease (TED) is commonly associated with Graves’ disease, often occurring concurrently or shortly after its diagnosis. However, atypical presentations, such as TED emerging years after thyroid function stabilization, are rare and can pose diagnostic challenges. These delayed cases may lead to misdiagnosis or delayed treatment, underscoring the importance of maintaining clinical vigilance, even in patients with long-term thyroid stability.

We describe a patient with a remote history of Graves’ disease who developed TED years after achieving thyroid stability on hormone replacement therapy following radioactive iodine therapy. She presented with unilateral strabismus and diplopia, prompting further evaluation. While TED was not initially suspected, and despite thyroid function tests within normal limits, significantly elevated thyrotropin receptor antibodies (TRAb) suggested disease reactivation. Progressive orbital signs and symptoms confirmed the diagnosis of TED, and treatment led to both clinical improvement and a subsequent decline in TRAb levels, reinforcing the role of antibody monitoring in atypical cases.

This case highlights the need for ongoing awareness of TED in patients with prior Graves’ disease, even long after thyroid function normalization, particularly with atypical presentations lacking classic TED findings. The marked elevation of TRAb served as a key biomarker for disease reactivation, emphasizing the value of antibody monitoring in late-onset TED cases. Early recognition and appropriate management are crucial to preventing complications and improving patient outcomes.

## Introduction

Overview of thyroid eye disease

Thyroid eye disease (TED), also known as Graves’ ophthalmopathy, is a complex autoimmune-mediated inflammatory disorder occurring in patients with hyperthyroidism due to Graves’ disease. It may also develop in euthyroid or hypothyroid patients or other thyroid autoimmune disorders [1]. TED is driven by thyrotropin receptor antibodies (TRAb), which not only target the thyroid gland but also cross-react with thyroid-stimulating hormone (TSH)-expressing orbital target cells (e.g., fibroblasts and pre-adipocytes) [2]. An extensive series of patients with recent onset of Graves’ disease found that 73.7% had no ocular involvement, 20.2% had mild and inactive TED, 5.8% had moderate-to-severe TED, and 0.3% had sight-threatening disease [3]. In this study, ocular involvement referred to any clinical signs of TED, ranging from mild inactive symptoms to sight-threatening complications. The classic clinical features of TED include eyelid retraction and proptosis. Diplopia often develops in more advanced cases as extraocular muscle involvement progresses. Other symptoms include dry eyes, redness, eyelid swelling, and retro-orbital pain. Vision loss due to optic nerve compression may occur in rare cases [1-5]. The initial active inflammatory phase, typically lasting six months to two years, is followed by progression to a chronic noninflammatory phase associated with fibrosis of the affected tissues [5]. Given the spectrum of signs and symptoms and varying levels of severity, the diagnosis can be challenging.

Atypical thyroid eye disease presentations

Atypical TED refers to presentations where the clinical features, timeline, or associations deviate from the common bilateral and hyperthyroid-associated form. Examples include delayed onset of TED following the initial diagnosis of Graves’ disease, atypical symptoms without the classic signs of TED, unilateral disease, severe TED without identifiable risk factors, occurrence in euthyroid or hypothyroid patients, optic neuropathy without proptosis, and presentation in the context of non-thyroid autoimmune conditions or orbital pathology. Atypical TED cases can result in diagnostic and treatment delays. Hence, a thorough evaluation of suspected TED is essential for diagnosis and timely referral to specialists for determination of optimal disease management, especially during the active phase of the disease, where treatment focuses on reducing inflammation and preventing long-term complications.

## Case presentation

Patient demographics

The patient is a 64-year-old nonsmoking female who developed new-onset binocular diplopia. She had a remote history of thyrotoxicosis due to Graves’ disease 27 years earlier, treated with radioactive iodine (RAI) and a subsequent subtotal thyroidectomy for a benign adenomatous nodule. She had no prior history of TED. Historical clinical records from that time were unavailable, but she remained clinically stable on thyroxine replacement with regularly monitored thyroid function tests.

She reported a long-standing history of intermittent strabismus characterized by occasional outward deviation of her right eye, typically occurring with eye fatigue and self-corrected with visual focus. This symptom reportedly began after RAI therapy but was infrequent, non-progressive, and without functional consequences. TED had not been suspected or identified during routine ophthalmic evaluations. At the time of the current presentation, her thyroid function was within normal limits, and there were no other changes in her systemic health.

Presenting complaint and clinical findings

Approximately two months prior to presentation, the patient developed intermittent binocular diplopia and difficulty focusing. These symptoms gradually progressed to persistent diplopia and right eye strabismus, no longer correctable with voluntary effort. No signs of orbital inflammation, such as redness, swelling, proptosis, pain, or vision loss, were present at the time of her initial evaluation by her ophthalmologist, who ordered emergent head and neck magnetic resonance imaging and angiography (MRI/MRA) to rule out a possible third nerve palsy or other cranial pathology. The imaging studies were negative for vascular or space-occupying lesions or significant orbital changes, and she was referred to a strabismus surgeon.

Over the subsequent two months, her symptoms progressed to include left eye proptosis with significant restriction of upward gaze, right eye exotropia with mild secondary hypertropia, persistent horizontal diplopia, and bilateral upper eyelid retraction. Based on this progression, the strabismus surgeon diagnosed TED, supported by an elevated TRAb level of 10 IU/L (normal range: 0.00-1.75 IU/L), and referred the patient to a TED clinic for further evaluation and management.

Diagnostic challenges

Her ophthalmologist dismissed the possibility of TED since her Graves’ disease history was so remote and she had no prior diagnosis of TED. Furthermore, other orbital conditions were suspected given her presentation with unilateral strabismus in the absence of classic TED symptoms (proptosis, pain, redness). This case reinforces the utility of TRAb testing in patients with strabismus who have a history of Graves’ disease to confirm the possibility of TED and avoid misattribution of strabismus to other causes.

Thyroid eye disease clinic evaluation and management

Examination by the Thyroid Eye Disease Specialty Team

Initial evaluation at the tertiary level included separate appointments with members of the TED specialty team, including an ophthalmologist, an oculoplastic surgeon, and an endocrinologist for examination, baseline photographs, and review of test results. The ophthalmic examination showed best-corrected visual acuity of 20/20 bilaterally, bilateral upper eyelid retraction, and Hertel exophthalmometry measurements of 14 mm and 17 mm on the right and left sides, respectively. A strabismus exam showed an incomitant left hypotropia and exotropia with excyclotorsion, consistent with a restrictive strabismus. The clinical activity score (CAS) was 0.

Additional Laboratory Testing

Results of laboratory testing are summarized in Table 1. Thyroid function tests (TSH, T4, and T3) were within normal limits, with an increased thyroid-stimulating immunoglobulin (TSI) index of 1.5 and an elevated repeat TRAb level of 9.18 IU/L. Myasthenia gravis-related antibody testing was also performed to evaluate for overlapping autoimmune conditions and was found to be negative.

**Table 1:** Thyroid and related autoantibody test results TSH: thyroid-stimulating hormone, sensitive; T4: thyroxine, free; T3: triiodothyronine, total; TSI: thyroid-stimulating immunoglobulin; TRAb: thyrotropin receptor antibodies; MuSK: muscle-specific kinase autoantibody

Test	Value	Normal range
TSH	1.6 mIU/L	0.03-4.2 mIU/L
T4	1.7 ng/dL	0.9-1.7 ng/dL
T3	3.7 pg/mL	2.8-4.4 pg/mL
TSI	1.5 TSI index	≤1.3 TSI index
TRAb	9.18 IU/L	0.00-1.75 IU/L
MuSK	0.00 nmol/L	0.00-0.02 nmol/L

Imaging Studies

**Figure 1:**
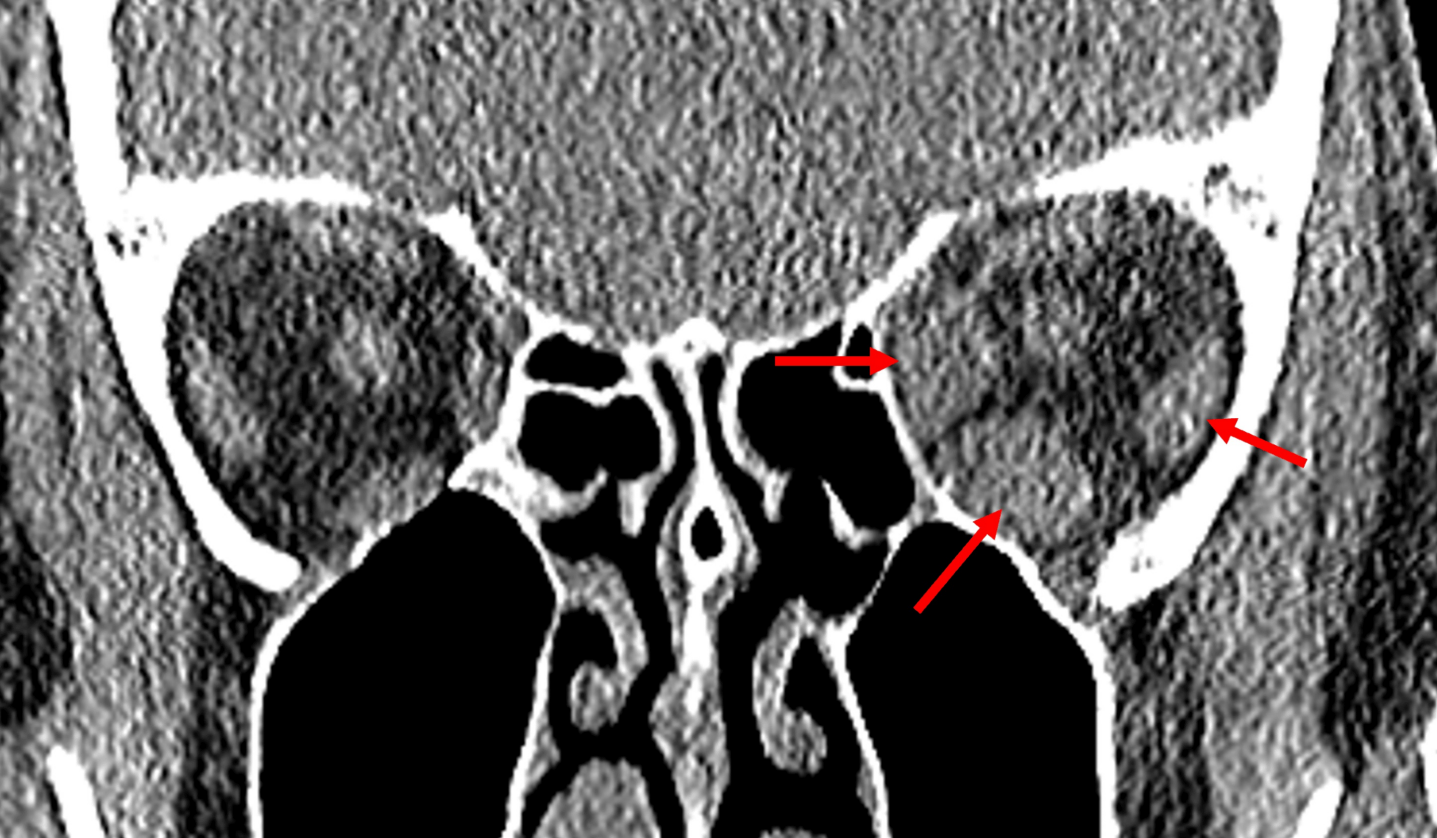
CT sinuses without contrast showing enlarged left medial, inferior, and to a lesser extent lateral rectus extraocular muscles (arrows) consistent with TED CT: computed tomography, TED: thyroid eye disease

CT imaging studies without contrast confirmed left proptosis with hyperplasia/hypertrophy of the left inferior and medial rectus muscles in a morphology compatible with thyroid-associated orbitopathy, with minimal enlargement of the dorsal left lateral rectus muscle and normal appearance of the right orbit, including extraocular muscles (Figure 1).

Management and Outcome

The TED clinical team reviewed the comprehensive findings to provide coordinated recommendations for treatment, taking into account disease activity, severity, and modifiable risk factors. Due to concerns regarding the potential adverse effects of teprotumumab, the patient opted for surgical management once it was confirmed that her TED had entered the inactive phase. Approximately six months after her initial evaluation, she underwent a left orbital decompression via a balanced two-wall approach to restore orbital balance and reduce proptosis for functional and cosmetic improvement. Approximately three months later, the patient underwent extraocular muscle surgery to correct strabismus, followed by later ocular adjustments to improve appearance and repair upper eyelid retraction. At her most recent follow-up, she had resolution of her binocular diplopia, except for mild torsional diplopia in eccentric gazes, which she managed with head positioning, and stable improvement in left proptosis. In addition to TRAb testing performed at the time of diagnosis, serial TRAb measurements were obtained throughout her management (Table 2).

**Table 2:** Serial TRAb values Normal range: 0.00-1.75 IU/L TRAb: thyrotropin receptor antibody

Variable	TRAb value (IU/L)	Interpretation
Initial test (Sep 17, 2021)	10	High
At TED clinic (Oct 19, 2021)	9.18	High
Follow up 1 (Jul 22, 2022)	5.98	High
Follow up 2 (Oct 27, 2023)	2.83	High
Follow up 3 (Dec 16, 2024)	2.29	High

**Figure 2:**
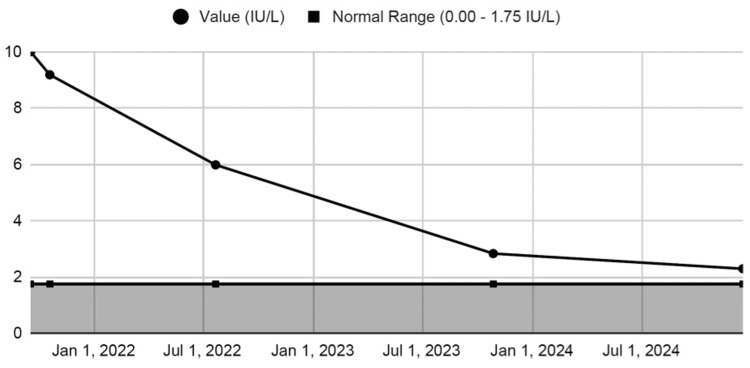
Trend in TRAb levels over the course of management TRAb: thyrotropin receptor antibody

As shown in Figure 2, initial TRAb levels were markedly elevated and demonstrated a progressive decline over time, correlating with the patient's clinical improvement.

## Discussion

Review of atypical presentation

This case is notable for its markedly delayed onset of TED in a euthyroid patient with a remote history of Graves' disease, initially presenting with diplopia but lacking other features of classic TED. Recognizing atypical presentations is crucial for diagnosis and effective management. Had TED been considered in the differential diagnosis during her initial presentation, referral to TED specialists would have occurred sooner, potentially limiting the progression and severity of her disease through early intervention.

"Quiet TED" subgroup

This case aligns with a subgroup of patients known as "quiet TED," characterized by the absence of active disease signs despite the presence of fibrotic sequelae and disease progression, challenging the classic disease paradigm. In a single-center study of a small cohort of 19 cases [6], patients primarily presented with diplopia (100%) and proptosis (63%). Most had a prior Graves' disease diagnosis and were euthyroid at TED onset. The disease was asymmetrical in 42% of the cases. In addition, the CAS was low at presentation; yet, 85% had moderate-to-severe disease. Hence, the unique expression of this TED patient subgroup poses significant diagnostic challenges, which may delay accurate identification and intervention.

Role of TRAb for diagnosis and monitoring of the disease

TRAb is central to the pathogenesis of Graves' disease and TED and is routinely used for diagnostic testing. It is often ordered in conjunction with a test for TSI, a specific subtype of TRAb that stimulates the TSH receptor, during the initial assessment of hyperthyroidism. Compared to TSI, TRAb tests are more widely available, less costly, and have shorter turnaround times, making TRAb an ideal biomarker for measurement of disease activity. A recent retrospective review demonstrated the utility of serial TRAb measurements in managing individual patients [7], concluding that such testing enables risk stratification, informs prognosis, guides treatment options, and helps prevent more severe manifestations by monitoring disease activity.

Humoral and cellular immune factors contribute to the sudden elevation of antibody titers. In patients with pre-existing antibodies, this response resembles an anamnestic antibody reaction, triggered by re-exposure to an antigen or a structurally similar protein. Upon such exposure, memory lymphocytes mount a rapid antibody response [8], a mechanism underlying phenomena like vaccine boosters and delayed hemolytic transfusion reactions. Similarly, fluctuating levels of pathogenic antibodies against self-antigens, such as TRAb, may result from immune memory responses, potentially triggering the reactivation of autoimmune disease.

Although TED often progresses gradually, timely diagnosis and management are crucial for patients with atypical presentations experiencing rapid progression to severe symptoms [9]. This case illustrates the role of TRAb testing as a critical biomarker for both the early detection of TED and monitoring ongoing disease activity.

Risks for thyroid eye disease development and reactivation

As observed in this case, TED significantly impairs the quality of life of affected individuals due to its debilitating effects [10], emphasizing the importance of identifying modifiable risk factors and recognizing them early for intervention and management to help limit progression to more severe disease. Risk factors for the development or deterioration of TED include elevated TRAb, female gender (although men who develop TED have more severe disease), genetic susceptibility, ancestry, other autoimmune disorders, pregnancy, and environmental factors, particularly tobacco smoking and RAI therapy [11-13]. Physical and emotional stress can affect thyroid hormone levels, which may cause or exacerbate Graves' disease and TED symptoms [14]. Additional factors associated with flare-ups, although rare, should also be considered, such as recent systemic infections that can lead to increased inflammation and immune system responses, which may exacerbate autoimmune conditions [15]. Similarly, flare-ups of autoimmune disease, including rare cases of TED reactivation, have been reported following vaccination [16-20]. Recently, a small series of cases of alemtuzumab-induced TED (AI-TED) have been reported [21]. Compared to conventional TED, AI-TED may present with greater severity many months after Graves' disease. Increased awareness of TED risk factors and prompt recognition of disease development or reactivation may guide treatment interventions and provide opportunities for immunotherapy during the active phase of disease [22].

Reactivation of TED is defined by the recurrence of inflammatory signs and symptoms after a period of stability lasting at least six months. Although uncommon, the recurrence of TED is not as rare as previously believed. Research indicates a recurrence rate of 15.7%, with most reactions occurring within the first 10 years of the initial episode [23]. Triggering events were identified in nearly 30% of cases and included smoking, fluctuating thyroid levels, stress, pregnancy, and periocular surgery (including cataract surgery and blepharoplasty). A definite trigger in this case was uncertain; however, several factors may have contributed to the patient's markedly elevated TRAb level, a key biomarker for disease reactivation. These include recent completion of the Pfizer-BioNTech COVID-19 (BNT162b2) and Zoster Vaccine Recombinant (SHRINGRIX) vaccination series within a few months of symptom onset, as well as significant worsening of symptoms coinciding with the recent death of a parent. Hence, the puzzling cause of this markedly delayed TED onset in a euthyroid patient with no history of smoking underscores the complexity of this disease and the importance of recognizing atypical presentations.

Delayed reactivation of thyroid eye disease

While historically considered rare, recent case series and studies highlight that late recurrences, defined as active orbitopathy after five years of quiescence, warrant greater clinical awareness. A few cases of "quiet TED" had a delayed onset [6], but the exact number of years was not specified. Other series have reported that most recurrences occur within 10 years of the initial episode [13,23]; however, cases of TED onset or reactivation documented more than 20 years later are rare. Recent reports have expanded this timeline, including a 2025 case series that identified TED reactivation at 20, 34, and 40 years post-initial TED [24]. Another frequently cited case by Manzouri et al. described TED onset 14 years after thyrotoxicosis [25]. Large prospective cohort studies, such as Stokland et al.'s 25-year follow-up, have identified late-onset TED occurring in approximately 10% of patients, although individual cases occurring more than 20 years after initial presentation were not detailed in this report [26].

This case report contributes valuable insight into the understanding of delayed-onset TED following thyroid function stabilization in patients with a history of Graves' disease. The 27-year latency observed in this case challenges historical assumptions regarding the self-limited course of TED. It highlights potential diagnostic pitfalls, as clinicians may overlook TED in patients with a remote history of thyroid disease. Furthermore, this case supports emerging evidence that TED can persist subclinically for decades, with disease flares potentially triggered by various risk factors. These findings underscore the importance of clinical vigilance and the utility of TRAb monitoring for detecting TED reactivation, even in the absence of overt thyroid dysfunction.

Treatment and management

The management of TED varies based on disease severity, as outlined in the American Thyroid Association and the European Thyroid Association consensus statements [27]. Traditionally, corticosteroids have been the primary treatment for moderate-to-severe TED, often combined with orbital radiotherapy in cases of optic neuropathy, alongside other supportive measures. However, there has been a growing shift towards medical therapy in recent years as newer therapeutic options have become available.

One of the most promising advancements is teprotumumab, a targeted therapy that demonstrates significant efficacy during the active phase of TED [23,28,29]. It was approved by the U.S. Food and Drug Administration in 2020 as the first medication specifically indicated for TED, offering the potential to stop disease progression and reverse its effects during the active phase [30-32]. Studies have examined its role in clinical practice, highlighting its effectiveness in TED management [23,33]. As a monoclonal antibody inhibitor of the insulin-like growth factor I receptor (IGF-IR), teprotumumab interferes with TSH receptor signaling, a key mechanism in TED pathogenesis [31-34]. Administered via intravenous infusions, it requires monitoring for effectiveness and adverse effects [35]. Teprotumumab has also been successfully used in patients with reactivated, refractory TED [36]. The expansion of clinical applications has spurred research into other molecularly targeted therapeutic approaches, such as tocilizumab, an IL-6 inhibitor, for steroid-resistant cases [37]. Batoclimab, an FcRn inhibitor, is another agent that has shown potential promise in preliminary studies [38].

For patients in the inactive phase of TED, when inflammation has subsided and fibrotic changes are typically present, surgical intervention remains the primary treatment option. While most procedures are planned electively in this phase, urgent surgical intervention may be necessary in cases of vision-threatening complications or severe corneal exposure. Management typically follows a staged approach: orbital decompression is done to address proptosis, followed by strabismus surgery to correct diplopia, and finally, eyelid surgery to repair retraction. Surgical success rates are high, offering significant relief and functional improvement [39,40].

## Conclusions

This case highlights the importance of recognizing TED as a potential delayed manifestation of Graves’ disease, even many years after the initial thyroid diagnosis. The atypical presentation underscores the need for vigilance in monitoring patients with a history of Graves’ disease, as well as the need to assess risk factors, which may be challenging to identify. This case emphasizes the utility of integrating biomarker trends with clinical assessment, diagnosis, and management of TED, particularly in atypical or late presentations. The elevated TRAb levels provided a valuable clue to the reactivation of autoimmune activity, correlating with the patient’s flare-up of TED. The subsequent decline in TRAb levels following treatment demonstrates the potential for antibody monitoring to guide therapeutic decisions and track disease progression and remission.

This report reinforces the importance of long-term follow-up in patients with Graves’ disease, even years after thyroid stabilization, to detect rare but clinically significant complications like delayed TED. Early recognition and appropriate management are crucial to preventing serious complications and optimizing outcomes. Further research and clinical trials are essential to refine treatment strategies and enhance our understanding of this complex disease.

## Appendices

**Table 3:** Glossary of terms/abbreviations and definitions

Term/abbreviation	Definition
Thyroid eye disease (TED)	An autoimmune inflammatory disorder affecting the orbit, commonly associated with Graves’ disease.
Thyrotropin receptor antibodies (TRAb)	Autoantibodies that bind to and stimulate or block the TSH receptor play a key role in the pathogenesis of Graves’ disease and TED.
Thyroid-stimulating immunoglobulin (TSI)	A subtype of TRAb that specifically stimulates the TSH receptor, promoting thyroid hormone production.
Thyroid-stimulating hormone (TSH)	A pituitary hormone that regulates thyroid gland function; measured to assess thyroid function.
Free thyroxine (T4)	The unbound, active form of the primary hormone produced by the thyroid gland.
Total triiodothyronine (T3)	A thyroid hormone involved in metabolism regulation; total includes both bound and unbound forms.
Radioactive iodine (RAI)	A treatment for hyperthyroidism that destroys thyroid tissue using RAI-131.
Clinical activity score (CAS)	A standardized score used to assess the activity of TED based on clinical signs and symptoms.
Muscle-specific kinase (MuSK)	An autoantibody tested to evaluate for myasthenia gravis, a differential diagnosis in patients with ocular symptoms.
Insulin-like growth factor I receptor (IGF-IR)	A receptor involved in TED pathogenesis; target of teprotumumab therapy.
Alemtuzumab-induced thyroid eye disease (AI-TED)	A form of TED triggered by the immunosuppressive drug alemtuzumab, often more severe.
Neonatal Fc receptor (FcRn)	A receptor involved in antibody recycling; targeted by novel therapies like batoclimab for autoimmune diseases.
Interleukin-6 (IL-6)	A cytokine involved in immune responses; targeted by agents like tocilizumab in steroid-resistant TED cases.
Magnetic resonance imaging (MRI)	A non-invasive imaging technique that uses magnetic fields and radio waves to visualize internal structures.
Magnetic resonance angiography (MRA)	A specialized MRI technique that provides detailed images of blood vessels.
Computed tomography (CT)	An imaging method that uses X-rays to create detailed cross-sectional views of the body, commonly used to assess orbital anatomy.
U.S. Food and Drug Administration (FDA)	The United States agency responsible for regulating drugs, medical devices, and treatments, including approval of TED therapies.
Exophthalmometry	A clinical method to measure the degree of forward protrusion (proptosis) of the eye, often using a Hertel exophthalmometer.
Diplopia	The perception of double vision, where a single object appears as two distinct images, common in strabismus and TED.
Strabismus	Misalignment of the eyes, often resulting in diplopia (double vision). Can be restrictive (from fibrosis) in TED.
Hypotropia	A form of strabismus where one eye is deviated downward.
Exotropia	A form of strabismus in which one or both eyes turn outward, away from the nose; may be intermittent or constant.
Excyclotorsion	Outward rotation of the eye around its visual axis; seen in orbital muscle involvement.
Euthyroid	A state in which thyroid hormone levels are within normal limits.
Fibrosis	Thickening and scarring of connective tissue, often a late-stage feature in TED affecting extraocular muscles.
Orbital decompression	A surgical procedure to relieve pressure in the eye socket, often performed to correct proptosis in TED.
Teprotumumab	A monoclonal antibody targeting IGF-IR used for treating active TED.
Thyroid function tests	Blood tests measuring TSH, T3, and T4 levels to evaluate thyroid status.
Thyroid autoantibodies	Immune proteins (e.g., TRAb, TSI) that target thyroid-related tissues; used in diagnosis and monitoring.
